# Finding Ponce de Leon’s Pill: Challenges in Screening for Anti-Aging Molecules

**DOI:** 10.12688/f1000research.7821.1

**Published:** 2016-03-29

**Authors:** Surinder Kumar, David B. Lombard

**Affiliations:** 1 Department of Pathology, University of Michigan, Ann Arbor, MI, 48109, USA; 2 Institute of Gerontology, University of Michigan, Ann Arbor, MI, 48109, USA

**Keywords:** aging, anti-aging medicine, age-related diseases

## Abstract

Aging is characterized by the progressive accumulation of degenerative changes, culminating in impaired function and increased probability of death. It is the major risk factor for many human pathologies – including cancer, type 2 diabetes, and cardiovascular and neurodegenerative diseases – and consequently exerts an enormous social and economic toll. The major goal of aging research is to develop interventions that can delay the onset of multiple age-related diseases and prolong healthy lifespan (healthspan). The observation that enhanced longevity and health can be achieved in model organisms by dietary restriction or simple genetic manipulations has prompted the hunt for chemical compounds that can increase lifespan. Most of the pathways that modulate the rate of aging in mammals have homologs in yeast, flies, and worms, suggesting that initial screening to identify such pharmacological interventions may be possible using invertebrate models. In recent years, several compounds have been identified that can extend lifespan in invertebrates, and even in rodents. Here, we summarize the strategies employed, and the progress made, in identifying compounds capable of extending lifespan in organisms ranging from invertebrates to mice and discuss the formidable challenges in translating this work to human therapies.

## Introduction

Aging is characterized by molecular, cellular, and organismal changes that culminate in the inability of an organism to maintain physiological integrity ^1^. In humans, aging is associated with a greatly increased predisposition to a wide variety of diseases, including cancer, type 2 diabetes (T2D), neurodegeneration, and cardiovascular disease, leading to increased morbidity and mortality ^1, 2^. The long-term objective of aging research is to develop interventions that can delay the onset of age-associated diseases and promote longevity. With this goal, research in biogerontology is focused on elucidating basic mechanisms of aging. Current evidence suggests that many of these mechanisms are conserved among eukaryotes, from yeast to mammals.

**Figure 1.**
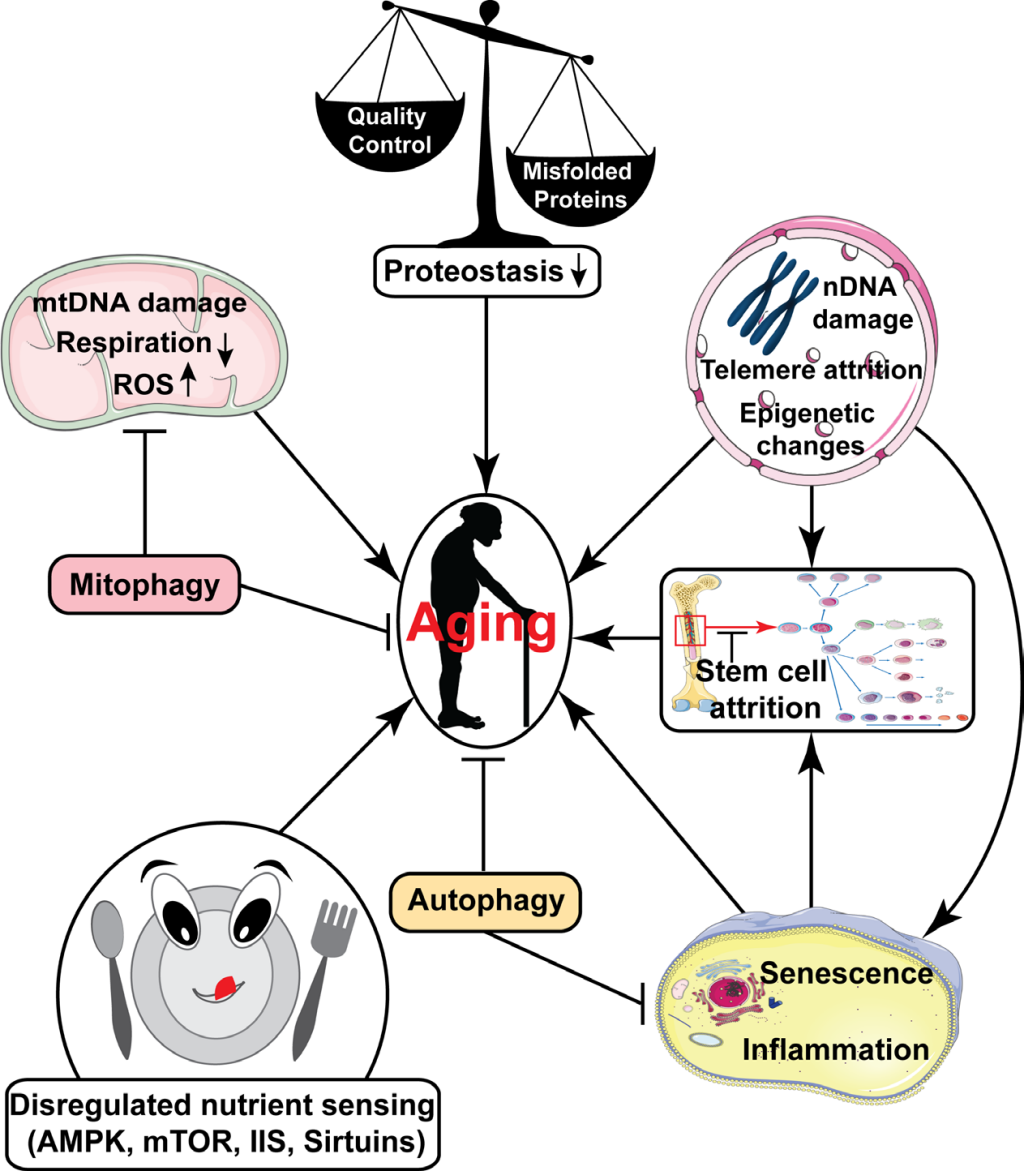
Summary of various factors that may contribute to aging. Dysregulation of nutrient-sensing pathways, mitochondrial dysfunction, loss of proteostasis, stem cell attrition, accumulated DNA damage, reduced autophagy, accumulation of senescent cells, and increased sterile inflammation are some important pathways thought to drive aging ^1^.

In recent decades, work in diverse organisms has identified cellular signaling pathways that modulate the aging rate ^3, 4^. Many of these pathways normally function to sense the nutritional status of the organism ( Figure 1) and initiate signaling cascades that modulate specific inter- and intra-cellular pathways and alter target cell physiology accordingly ^2^. These nutrient-sensing pathways, which include insulin and insulin-like growth factor (IGF) signaling (IIS) ^5^, target of rapamycin (mTOR) signaling ^6^, adenosine monophosphate (AMP)-activated protein kinase (AMPK) signaling ^7^, and sirtuins ^8^, coordinate cellular growth- and metabolism-related processes and integrate them with levels of nutrients, energy, growth factors, and stress. When nutrient levels and growth cues are reduced, signaling through these pathways is altered. Genetic or, in some cases, pharmacologic manipulation of these pathways can lead to lifespan extension, whereas their age-associated dysregulation may contribute to organismal senescence.

Dietary restriction (DR), a dietary regimen involving either a reduction in overall calorie ingestion without malnutrition or diminished intake of specific dietary components such as amino acids, is the best-characterized intervention that slows aging and delays disease in a wide range of species ^9, 10^. Molecular effectors implicated in mediating the remarkable effects of DR include these nutrient-sensing pathways ^9^. Initial evidence suggests that some of these same pathways may impact aging and disease in humans as well. For example, genetic variants in the *FOXO3A* gene, encoding a transcription factor downstream of IIS, have been linked to human longevity ^11– 16^. Individuals with Laron dwarfism have greatly reduced serum IGF1 levels and profound protection from T2D and cancer ^17^. Pharmacological interventions that partially mimic DR by modulating activities of these nutrient-sensing pathways have the potential to improve healthspan and promote longevity. For example, rapamycin, a specific inhibitor of mTOR, has been proposed to provoke some of the beneficial effects of DR under standard feeding and nutrient conditions ^18^. Similarly, a handful of other molecules such as metformin and resveratrol have been shown to modulate nutrient signaling and promote healthspan in multiple model organisms and are discussed in detail subsequently.

In addition to dysregulation of nutrient-sensing pathways, other conserved mechanisms implicated in the deleterious manifestations of aging include ( Figure 1) *i)* mitochondrial dysfunction, leading to impaired respiratory metabolism, increased generation of reactive oxygen species (ROS), as well as potentially other sequelae, *ii)* increased accumulation of DNA damage, induced by exogenous insults and endogenous hazards including DNA replication errors and ROS, *iii)* diminished proteostasis associated with increased protein misfolding and aggregation, *iv)* cellular senescence, contributing to tissue dysfunction, *v)* increased sterile inflammation, *vi)* stem cell attrition, and *vii)* epigenetic alterations ^1, 19^. For a more complete discussion of conserved aging mechanisms, the reader is referred elsewhere ^1^. Pharmacological agents targeting some of these changes represent candidate anti-aging drugs. In this review, we will provide an overview of pharmacological interventions with known or potential ability to delay aging and promote late-life health. First, we summarize the major contributions that studies in invertebrate model systems have made towards screening efforts to identify small molecule anti-aging drugs. Then we focus in depth on molecules currently under study for their potential to extend lifespan and delay disease. Finally, challenges in screening for new anti-aging drugs and in translating this work to humans will be discussed.

## Invertebrates as model systems to screen pro-longevity small molecules

Due to a variety of factors – notably including ease of genetic manipulation and a physiology similar to that of humans – the mouse has become the pre-eminent mammalian model organism in aging biology ^20^. However, in light of the high housing costs and relatively long lifespan of mice, large-scale unbiased screening to identify anti-aging medicines is not feasible in this organism. With the realization that many aging-related pathways are evolutionarily conserved, even among widely divergent species, short-lived invertebrate models have instead been employed for such screening. The nematode *Caenorhabditis elegans* – with its short lifespan of ~3 weeks, ease of culture and genetic manipulation, and well-characterized aging biology – represents a very attractive model system for chemical screening to identify compounds that modulate lifespan and age-related phenotypes. Indeed, several studies have identified a number of candidate anti-aging compounds using *C. elegans* as a model organism. To date, the most comprehensive small molecule lifespan screen using *C. elegans* was conducted by Petrascheck *et al.*, who evaluated 88,000 chemicals for their ability to enhance longevity ^21^. They identified 115 compounds that significantly increased worm lifespan. Interestingly, one of these displayed structural resemblance to human antidepressants that affect signaling by the neurotransmitter serotonin. They subsequently found that mianserin, a serotonin receptor antagonist used as an antidepressant in humans, extends *C. elegans* lifespan when administered at 50 μM, likely via mechanisms linked to DR ^21^. In an evaluation of 19 compounds with known effects on human physiology, Evason *et al.* reported that the anticonvulsants ethosuximide (dosed at 2 and 4 mg/mL), trimethadione (4 mg/mL), and 3,3-diethyl-2-pyrrolidinone (2 mg/mL) delayed age-related changes and increased *C. elegans* lifespan ^22^.

Using a bioinformatics approach to identify DR mimetics, Calvert *et al.* analyzed drugs that induce gene expression changes similar to those associated with DR and identified 11 small molecules with this property ^23^. Interestingly, among five drugs tested, four – rapamycin (administered at 10 μM), allantoin (250 μM), trichostatin A (100 μM), and LY-294002 (100 μM) – provoked increased lifespan and healthspan in wild-type (WT) *C. elegans*. Conversely, no longevity effects were observed in the *eat-2* mutant background, a genetic DR model, suggesting that the life-extending effects of these drugs may indeed occur via DR-related mechanisms ^23^.

A study by Alavez *et al.* reported that amyloid-binding compounds maintain protein homeostasis and extend lifespan in *C. elegans* ^24^. Exposure of WT worms to the amyloid-binding dye Thioflavin T (ThT) at either 50 or 100 μM throughout adulthood increased median lifespan by 60% and maximal lifespan by 43–78% ^24^. ThT treatment reduced Aβ-aggregation and preserved muscle integrity in *C. elegans* models of Alzheimer’s disease (AD), resulting in a decreased proportion of paralyzed worms. ThT administration also suppressed the toxicity associated with metastable proteins in mutant worms ^24^. ThT-mediated suppression of protein aggregation and lifespan extension depended upon molecular chaperones, autophagy, proteosomal function, the proteostasis regulator heat shock factor 1 (HSF-1), and the stress resistance and longevity transcription factor SKN-1 ^24^. Compounds with structural similarity to ThT also extended worm lifespan by up to 40%, but at significantly lower concentrations than ThT. Moreover, exposure to other protein-aggregate-binding compounds like curcumin (100 μM) and rifampicin (10–100 μM) extended worm lifespan by up to 45% ^24^. These results highlight the importance of proteostasis in worm healthspan and lifespan, and provide further impetus for the development of interventions capable of maintaining proteostasis to suppress aging and age-related diseases.

The National Institute of Aging has recently sponsored a pharmacological intervention program using *Caenorhabditis* as a model system, analogous to similar ongoing efforts in the mouse. The *Caenorhabditis* Intervention Testing Program (CITP) is a multi-institutional effort aimed at identifying compounds with the ability to extend lifespan and enhance healthspan, using multiple *Caenorhabditis* species and multiple strains of *C. elegans*. The identification of compounds that are effective in genetically diverse worm populations may accelerate the discovery of interventions that can extend lifespan/healthspan in other species, potentially including humans.

The fruit fly *Drosophila melanogaster* represents another model suitable for the screening of anti-aging compounds ^25^. A wide variety of genetic strains of *D. melanogaster* are available, with different mean lifespans, useful for validation of compound efficacy across multiple genetic backgrounds. Similar to *C. elegans*, *Drosophila* has a short lifespan, and the many genetic tools available in this organism facilitate mechanistic study of lead compounds ^25^. The first study reporting lifespan extension in *Drosophila* by administration of a drug was performed by Kang *et al.,* who showed that feeding *Drosophila* 4-phenylbutyrate at 5–10 mM – a drug with multiple activities, including histone deacetylase inhibition – significantly increased both median and maximum lifespan without negative impacts on locomotion, stress resistance, or reproduction ^26^. A more recent study described the screening of protein kinase inhibitors for effects on *Drosophila* lifespan ^27^. Among the 80 inhibitors tested in this study, 17 significantly increased *Drosophila* lifespan without affecting food intake or consumption, indicating that the effects of these inhibitors on *Drosophila* lifespan do not involve DR ^27^. In this regard, a recent study by Slack *et al.* reported that attenuation of RAS-Erk-ETS signaling results in reduced IIS and provokes lifespan extension in *Drosophila* ^28^. Trametinib (1.56–15.6 μM), a highly specific MEK inhibitor that attenuates signaling downstream of RAS, can prolong median lifespan of female *Drosophila* by up to 12% (p=1.92 × 10 ^-10^), and at higher doses (156 μM), improves late-life survival ^28^. Trametinib administration was effective in promoting fly longevity even when administered to middle-aged animals. These and similar findings with other drugs – *cf*. extension of mouse lifespan by rapamycin treatment initiated in middle age, see below – raise the possibility that anti-aging medicines in humans might be effective even when administered to older individuals, thus avoiding potential developmental side effects of these drugs.

## Compounds that modulate aging and age-associated phenotypes in mammals

### The mTOR inhibitor rapamycin

mTOR is a conserved serine/threonine kinase that senses and responds to nutrient availability, growth factors, and environmental stress and plays a key role in triggering growth ^6, 29^. In multicellular eukaryotes, mTOR exists in two distinct multi-protein complexes, mTORC1 and mTORC2, distinguished by their association with regulatory-associated protein of mTOR (RAPTOR) and rapamycin-insensitive companion of mTOR (RICTOR), respectively ^30, 31^. Rapamycin forms a complex with the FKBP12 protein, which binds to mTORC1 and inhibits its activity ^32^. Importantly, chronic treatment with rapamycin also inhibits mTORC2 ^33^. mTORC1 activity is regulated by nutrients (glucose and amino acids), cytokines, hormones (insulin or IGF1), energy (ATP levels), and oxidative stress via PI3K, AKT, and AMPK signaling ^6^. Key downstream mediators of mTORC1 signaling are pathways that control cell growth, proliferation, stress response, and autophagy ^29, 34^. mTORC1, therefore, critically integrates cellular growth and maintenance with nutrient availability, hormonal cues, and other environmental stimuli.

A number of studies have established a link between mTOR signaling pathways and longevity in organisms ranging from yeast to mammals. Inhibition of mTOR signaling by genetic or pharmacologic means extends lifespan in yeast ^35– 37^, nematodes ^38, 39^, fruit flies ^40^, and mice ^33, 41– 47^. Likewise, genetic deletion in mice of the downstream mTORC1 effector, S6 kinase 1, increases oxidative metabolism, protects against age- and diet-induced obesity, and increases female lifespan ^47, 48^. Consistently, enhanced activity of the mTORC1 target 4E-BP1 in skeletal muscle results in increased oxidative metabolism and protects mice from diet- and age-induced metabolic dysfunction ^49^.

In a landmark study, NIA’s Interventions Testing Program (ITP) showed that treatment of a genetically heterogeneous mouse stock with the mTOR inhibitor rapamycin (administered at 14 mg/kg food; 2.24 mg/kg body weight/day) initiated at either 9 months or 20 months of age extended lifespan in both sexes ^43, 50^. A follow-up study demonstrated that the increase in mouse lifespan induced by rapamycin is dose and sex dependent. At a given chow concentration of rapamycin, female mice showed a greater increase in lifespan than did males, which correlated with higher blood levels of rapamycin achieved in females relative to males ^51^. Rapamycin treatment induced entirely distinct gene expression changes in males and females, implying the existence of sex-specific responses to mTOR inhibition ^51^. Furthermore, the expression patterns of xenobiotic-metabolizing enzymes in the livers of rapamycin-treated (14 mg/kg food) mice differed strikingly from those in DR-exposed animals at 12 months of age ^51^. Indeed, DR is less effective in lifespan extension when initiated later in life ^52– 54^, while rapamycin treatment extends the lifespan of mice, even when started in middle age ^43, 55^. Crucially, rapamycin-induced lifespan extension in mice has also been observed in diverse genetic backgrounds ^41, 42, 44, 56^.

Mechanisms of longevity extension by rapamycin remain a hotly debated topic in aging biology ^56, 57^. Rapamycin has anti-neoplastic properties ^58– 60^, and cancer is the major cause of death in most mouse strains that show rapamycin-mediated lifespan extension ^43, 61^. In this context, one plausible explanation for the extension of mouse lifespan by rapamycin is that this drug suppresses the onset and/or aggressiveness of lethal cancers. However, some investigators have reported that rapamycin also inhibits age-associated phenotypes besides neoplasia ^62, 63^, strongly suggesting that this drug has broader anti-aging effects. In contrast, a recent exhaustive study by Neff *et al.* claimed that the effects of rapamycin on aging phenotypes *per se* were quite limited ^56^. In this regard, conflicting observations have been made concerning the effects of rapamycin treatment in mouse models of AD ^64^. Long-term rapamycin treatment led to behavioral improvements in mouse AD models and induced an autophagy-mediated decrease in Aβ and hyperphosphorylated tau levels ^65, 66^. Conversely, rapamycin has been shown to promote Aβ production ^67, 68^ and led to an increase in Aβ-induced cell death ^69^.

Rapamycin has significant side effects – metabolic dysfunction, cataract, and testicular atrophy in particular – that may limit its long-term utility as an anti-aging treatment in humans ^70, 71^. Most importantly, due to the immunomodulatory effects of mTOR inhibitors, treatment of human patients with the rapamycin-like drug everolimus/RAD001 is associated with a higher incidence of infection in individuals with diseases such as cancer ^72, 73^ and tuberous sclerosis complex (TSC) ^74^. Conversely, a recent study showed that short-term administration of everolimus/RAD001 to healthy older individuals enhanced the immunological response to influenza vaccination, with modest side effects ^75^. Decreased influenza vaccine response is a major clinical challenge in older populations ^76^. These findings suggest that intermittent or short-term administration of rapamycin or other mTOR inhibitors might suppress certain functionally important effects of aging, such as poor immunization response, while avoiding the negative consequences associated with chronic use of these agents. A recent study in mice is consistent with this view, identifying an intermittent rapamycin administration regimen in mice that minimizes metabolic dysfunction, while maintaining chronic mTORC1 suppression in adipose tissue, though not in other tissues ^77^. It will be of great interest to evaluate the effects of such intermittent dosing regimens on a wide range of age-associated phenotypes and on lifespan.

### Metformin and other biguanides

Metformin, an oral biguanide antiglycemic agent, is the most widely used drug in the treatment of metabolic syndrome and T2D. Metformin’s mechanism of action is not completely understood and is likely to be multi-factorial. It was reported to decrease serum glucose levels by inhibiting respiratory chain Complex I in hepatocytes ^78^, resulting in reduced ATP production, leading to activation of the LKB1 and AMPK kinases, suppressing hepatic gluconeogenesis ^79, 80^. Metformin has been reported to activate AMPK in many other tissues, including adipose, skeletal muscle, heart, pancreatic β-cells, and hypothalamus with potential beneficial physiological effects in patients with T2D ^81, 82^. However, metformin also exerts important effects independent of AMPK and LKB1 ^83^, *e.g.* by antagonizing the action of glucagon ^84^. Recently, another AMPK-independent mechanism has been revealed for metformin. A study by Madiraju *et al.* showed that metformin non-competitively inhibits the redox shuttle enzyme mitochondrial glycerophosphate dehydrogenase, increasing the cytosolic redox state and decreasing the mitochondrial redox state ^85^. This suppresses hepatic gluconeogenesis by reducing the conversion of lactate and glycerol to glucose ^85^. Although metformin is currently approved for treatment of T2D, a large literature suggests efficacy of metformin against other conditions, particularly cardiovascular diseases and cancer ^78^. In this regard, a recent study demonstrated that metformin reduces tumorigenesis by inhibiting mitochondrial Complex I in cancer cells ^86^.

AMPK activation provokes longevity in flies and worms ^87, 88^. A number of studies suggest that metformin treatment can recapitulate some effects of DR. In this context, several studies have examined the effects of metformin and other biguanides on lifespan and reported a variety of outcomes. Metformin and other biguanides extend *C. elegans* lifespan in a dose-dependent manner ^89– 91^. The increase in *C. elegans* lifespan by metformin is mediated through inhibition of bacterial folate and methionine metabolism, which in turn alters methionine metabolism in the worm, resulting in reduced S-adenosylmethionine and increased S-adenosylhomocysteine levels ^89^. However, metformin apparently does not extend longevity in *D. melanogaster* ^92, 93^. Indeed, despite robust activation of AMPK, high doses of metformin actually decrease lifespan of both male and female flies ^93^, perhaps due to disruption of intestinal fluid homeostasis ^93^. However, metformin treatment suppressed age-related phenotypes in intestinal midgut stem cells ^94^ and also exerted beneficial effects in a fly obesity model ^95^. A recent study showed that metformin treatment causes a significant extension in mean and maximal lifespan in both sexes of the cricket *Acheta domesticus* ^96^.

Several studies have been performed in rodents to test the effects of metformin and other biguanides on lifespan; the outcomes have varied with genotype, sex, and dose and duration of treatment ^97^. Chronic treatment with metformin (100 mg/kg in the drinking water) enhanced the mean lifespan of cancer-prone HER-2/neu transgenic, outbred SHR, and inbred 129/Sv female mice by 8% (p<0.05), 37.8% (p<0.01), and 4.4% (p<0.05), respectively ^98– 100^. Metformin treatment also extended the maximum lifespan of HER-2/neu transgenic and outbred SHR female mice by 9% and 10.3%, respectively, while no effect was observed on maximal lifespan in inbred 129/Sv female mice ^98– 100^. Conversely, treatment of inbred 129/Sv male mice with a similar dose of metformin actually *reduced* mean lifespan by 13.4% ^100^. However, metformin treatment (2 mg/mL in drinking water) in a transgenic mouse model of Huntington disease (HD) prolonged male mean lifespan by 20.1% (p=0.017), but did not affect female survival ^101^. It has been reported that metformin treatment (100 mg/kg in the drinking water) of female outbred SHR mice initiated at 3 months of age induced a trend towards increased mean lifespan ^102^. Metformin treatment also postponed the onset of detectable tumors when started at young or middle ages, but not at old age ^102^. Neonatal metformin treatment of 129/Sv mice (100 mg/kg via subcutaneous injection) led to a 20% (p<0.001) increase in male mean lifespan and also slightly increased maximum lifespan by 3.5% ^103^. However, in females, the mean and maximum lifespan in metformin-treated groups were decreased by 9.1% and 3.8%, respectively ^103^. In a recent study by Martin-Montalvo *et al.*, male C57BL/6 mice supplemented with 0.1% metformin in the diet showed a 5.8% increase in mean lifespan (p=0.02, Gehan–Breslow survival test), whereas supplementation with 1% metformin was toxic and reduced mean lifespan by 14.4% ^104^. However, supplementation of B6C3F1 male mice with 0.1% metformin resulted in extension of mean lifespan only by 4.2% (p=0.064, Gehan–Breslow) ^104^. Treatment with another biguanide, phenformin (2 mg/mouse in 0.2 mL of drinking water), significantly reduced spontaneous tumor development in female C3H/Sn mice and prolonged mean lifespan by 21% or more (p<0.05) ^105, 106^ and maximum lifespan by 26% ^105^. Evaluation of the lifespan effects of metformin in mice by the ITP consortium is ongoing, and the results should be available soon.

In rats, buformin treatment (5 mg/rat in 1 mL of drinking water) led to a non-significant 7.3% increase in mean lifespan of female LIO animals, while phenformin (5 mg/rat in 1 mL of drinking water) had no effect ^105^. However, administration of both buformin and phenformin increased the maximum lifespan of female LIO rats by 5.5% and 9.8%, respectively ^105^. Treatment with metformin (300 mg/kg/day) did not increase either mean or maximum lifespan of male F344 rats ^107^. However, in the same report, a parallel group of male F344 rats exposed to DR also failed to exhibit lifespan extension ^107^, leaving the metformin results in this study somewhat inconclusive. Mechanistically, treatment with metformin has been proposed to mimic some effects of DR, in particular by increasing AMPK activity and also activating antioxidant responses, leading to a reduction in both oxidative damage accumulation and chronic inflammation ^104^.

Although no study has formally analyzed the effects of long-term metformin treatment on lifespan in healthy humans, randomized clinical trials of metformin showed beneficial effects on health and survival in overweight/obese patients with T2D, as shown by decreased incidences of cardiovascular disease and cancer and reduced overall mortality ^108– 110^. However, when combined with sulfonylurea, metformin *increased* the risk of diabetes-related death and all-cause mortality in a mixed group of non-overweight and overweight/obese individuals with T2D ^78, 108^. Consistent with these observations, a recent study by Bannister *et al.* reported that patients with T2D treated with metformin displayed improved survival compared to matched, non-diabetic controls, whereas those treated with sulfonylureas showed reduced survival ^111^.

Given the relatively promising rodent data, the hints that metformin might suppress cancer and other age-associated conditions in humans, and metformin’s relatively benign safety profile, there is great current interest in formally testing the ability of this drug to delay age-associated disease in humans ^112^. Indeed, the US Food and Drug Administration (FDA) recently approved a study termed Targeting Aging With Metformin (TAME) for the evaluation of metformin as an anti-aging drug. The TAME project will involve approximately 3000 participants between the ages of 70 years and 80 years who either already have one, two, or all three of the conditions: cancer, heart disease, or cognitive impairment or are at risk of developing them. The trial will take place at roughly 15 centers around the United States over 5–7 years, costing approximately $50 million ^113^. The goal of the study is to determine whether metformin can prevent the onset of age-associated disease. This landmark trial will represent the first testing of a candidate anti-aging compound in humans.

### Resveratrol and other sirtuin-activating compounds

The sirtuins are a family of NAD ^+^-dependent deacetylases/ADP-ribosyltransferases/deacylases implicated in regulating nutrient responses and numerous other aspects of cell biology ^8^. Overexpression of Sir2, the founding member of the sirtuin family, extends replicative lifespan in the budding yeast *Saccharomyces cerevisiae* by repressing the accumulation of extrachromosomal rDNA plasmids, promoting segregation of an undamaged proteome to the daughter cell, enforcing subtelomeric silencing, and perhaps other mechanisms ^114, 115^. Several, though not all, investigators have found that overexpression of sirtuins in worms and flies modestly increases lifespan in these organisms ^116– 123^. Interestingly, the Sir2 homolog Sir-2.1 can extend *C. elegans* lifespan in a manner independent of its deacetylase activity ^116^. Indeed, nicotinamide (NAM), a product of sirtuin activity, and its metabolite, 1-methylnicotinamide (MNA), are capable of extending worm lifespan, potentially by inducing transient ROS signaling ^116^. In mammals, SIRT1 is the closest Sir2 homolog; overexpression of this protein in the brain (but not the whole organism) extends lifespan ^124^, probably by enhancing hypothalamic function during aging ^125^. Global overexpression of another sirtuin, SIRT6, extends mouse lifespan in males specifically, at least in part via suppression of lung cancer, a major cause of death in males of the mouse stock used ^126, 127^. SIRT2 overexpression stabilizes levels of the mitotic checkpoint protein BubR1 in progeroid *BubR1 ^H/H^* mice and extends both median and maximum lifespan in male mice of this strain ^128^. No information is available concerning the potential effects of chronic SIRT2 overexpression in WT animals. Accumulating evidence suggests that NAD ^+^ levels may decline during aging, impairing sirtuin activity, and that the ability of sirtuin overexpression to increase lifespan partially counters this effect by maintaining sirtuin function in the face of a diminished NAD ^+^ pool in older organisms ^129^.

Resveratrol and certain other polyphenols were originally identified as Sir2/SIRT1 activators that extended the average and maximal lifespan of yeast ^130^. It is important to note that resveratrol is a highly promiscuous drug and exerts functionally important effects on many cellular targets ^131^. Treatment of worms and flies with resveratrol (dosed at 100 μM in worms and 10–100 μM in flies) has also been reported to extend lifespan, dependent on the presence of functional Sir-2.1 and dSir2, respectively ^132^. However, a study by Bass *et al.* claimed that resveratrol treatment (1–1000 μM) had no significant effects on *Drosophila* lifespan ^133^. The same study also reported that resveratrol treatment at 100 μM induced only a slight and sporadic increase in *C. elegans* lifespan in both WT and *sir-2.1* mutant animals, suggesting that these small increases in *C. elegans* lifespan induced by resveratrol may be *Sir-2.1* independent ^133^. Resveratrol protects worms from oxidative stress, radiation-induced damage, and amyloid toxicity ^134– 136^ and also induces radioprotection in flies ^137^. Resveratrol treatment increases mean and maximum lifespan in the honeybee ^138^ and the short-lived fishes *Nothobranchius furzeri* and *Nothobranchius guentheri* ^139– 141^.

It was reported that resveratrol and other sirtuin-activating compounds (STACs) activate Sir2/SIRT1 allosterically ^130^. However, other groups have found that these compounds were unable to enhance SIRT1 activity towards native peptides *in vitro* ^142, 143^. In this context, it has been suggested that increased SIRT1 activity induced by resveratrol depends on the presence of a non-native fluorophore conjugated to the peptide sequence originally used in screening for SIRT1 activators ^142, 143^. Recent reports, however, have shown that resveratrol and other STACs directly bind to SIRT1 and allosterically enhance its deacetylase activity towards non-tagged peptide substrates ^144, 145^. Resveratrol has also been reported to inhibit the catalytic activity of human tyrosyl transfer-RNA (tRNA) synthetase (TyrRS), resulting in its nuclear translocation and stimulation of NAD ^+^-dependent activation of poly (ADP-ribose) polymerase 1 (PARP1) ^146^. PARP1 plays important roles in both DNA repair and transcription ^147^.

In mice, resveratrol is protective against some damaging effects of high-fat/high-calorie diets ^148– 151^, substantially reduces the growth and development of multiple types of cancers ^152– 154^, and delays or prevents the onset of AD ^155, 156^. Moreover, in rodents and humans, resveratrol is protective against both type 1 diabetes and T2D ^157, 158^ and cardiovascular disease ^159^ and possesses anti-inflammatory ^160^ and anti-viral activities ^161^. Resveratrol supplementation (either at 0.016–0.1% of diet or 25 mg/kg/day) has been reported to increase lifespan in mouse models of obesity ^148^, AD ^162^, HD ^163^, and amyotrophic lateral sclerosis ^164, 165^. Resveratrol treatment (2–8 mg/kg/day) increases the lifespan of LPS-treated mice ^166^ and attenuates catecholamine-induced mortality in obese rats (20 mg/kg/day) ^167^. Furthermore, resveratrol (10 mg/mL, intraperitoneal injection) prolongs survival in a mouse model of sepsis-induced acute kidney injury and restores renal microcirculation ^168^. Resveratrol administration (18 mg/kg/day in the diet) also improves survival in a rat hypertension model ^169^. Importantly, however, resveratrol treatment (100–1200 mg/kg food) does not increase lifespan in normal chow-fed mice ^50, 170, 171^. Resveratrol supplementation induces gene expression changes in several tissues that resemble those associated with calorie restriction in mice ^171, 172^.

In humans, 30-day resveratrol supplementation (150 mg/day) in obese men induced metabolic changes, including reductions in sleeping and resting metabolic rate, intrahepatic lipid content, circulating glucose levels, inflammatory markers, and systolic blood pressure ^173^. Skeletal muscle from resveratrol-treated objects displayed increased AMPK activity, increased SIRT1 and PGC-1α protein levels, and improved mitochondrial respiration of fatty acids ^173^. In contrast, 12 weeks’ supplementation with resveratrol (75 mg/day) in non-obese, postmenopausal women with normal glucose tolerance induced no apparent change in body composition, insulin sensitivity, resting metabolic rate, plasma lipids, or inflammatory markers ^174^. Moreover, resveratrol supplementation had no effect on its putative molecular targets, including AMPK, *SIRT1*, *NAMPT*, and *PPARGC1A*, in either skeletal muscle or adipose tissue ^174^.

An important recent study by Cai *et al.* demonstrated a non-linear dose response for the protective effects of resveratrol in humans and mice ^175^. When co-administered with high-fat diet (HFD), low-dose resveratrol (~0.07 mg/kg/day) appeared to be more efficacious than high-dose (14 mg/kg/day) in reducing adenoma number and decreasing overall tumor burden in *Apc ^min^* mice, a model of intestinal carcinogenesis. Interestingly, female mice on the lower dose of resveratrol exhibited significantly higher expression and activation of AMPK in intestinal mucosa than those in the high-dose group ^175^. Consistently, human colorectal tissues exposed to low dietary concentrations (0.01 to 0.1 μM) of resveratrol *ex vivo* displayed rapid AMPK activation and increased autophagy at low concentrations and a less pronounced or even no effect at higher doses (1 to 10 μM) ^175^. This unusual effect may help rationalize the conflicting reports of resveratrol’s efficacies in humans, and future human studies using resveratrol must be designed with careful attention paid to dosage and serum levels and to a thorough assessment of effects on resveratrol’s putative molecular targets.

Other STACs have been synthesized and are reported to enhance healthspan and extend lifespan in mice. The STAC SRT1720 (100 mg/kg/day) has been reported to extend mean lifespan of adult male C57BL/6J mice fed a standard diet by 8.8% (p=0.096), and up to 21.7% (p=0.0193) on a HFD, without increasing maximal lifespan in either context ^176, 177^. SRT1720 treatment improved physiological parameters in HFD-fed animals, reducing liver steatosis, increasing insulin sensitivity, enhancing locomotor activity, and also inducing a gene expression profile similar to that associated with a standard diet ^176^. SRT1720 supplementation inhibited pro-inflammatory gene expression in liver and muscle of mice fed a standard chow diet and delayed the onset of age-related metabolic disease ^177^. Similarly, dietary supplementation (100 mg/kg) with SRT2104, another synthetic STAC, increased both mean and maximal lifespan of male C57BL/6J mice fed a chow diet by 9.7% (p<0.05) and 4.9% (p<0.001), respectively, and increased insulin sensitivity and motor coordination while reducing inflammation ^178^. Short-term treatment with SRT2104 preserves bone and muscle mass in an experimental atrophy model ^178^. These findings indicate that resveratrol and other STACs can exert beneficial effects on health, particularly in the context of HFD, and that some STACs can modestly extend lifespan under normal feeding conditions; however, additional studies are warranted to better evaluate their effects on longevity in females and other strains of mice. In this regard, there is great current interest in evaluating the effects of NAD ^+^ precursors as therapies for metabolic disease and candidate anti-aging drugs ^129^.

### Other potential candidate anti-aging drugs

**Figure 2.**
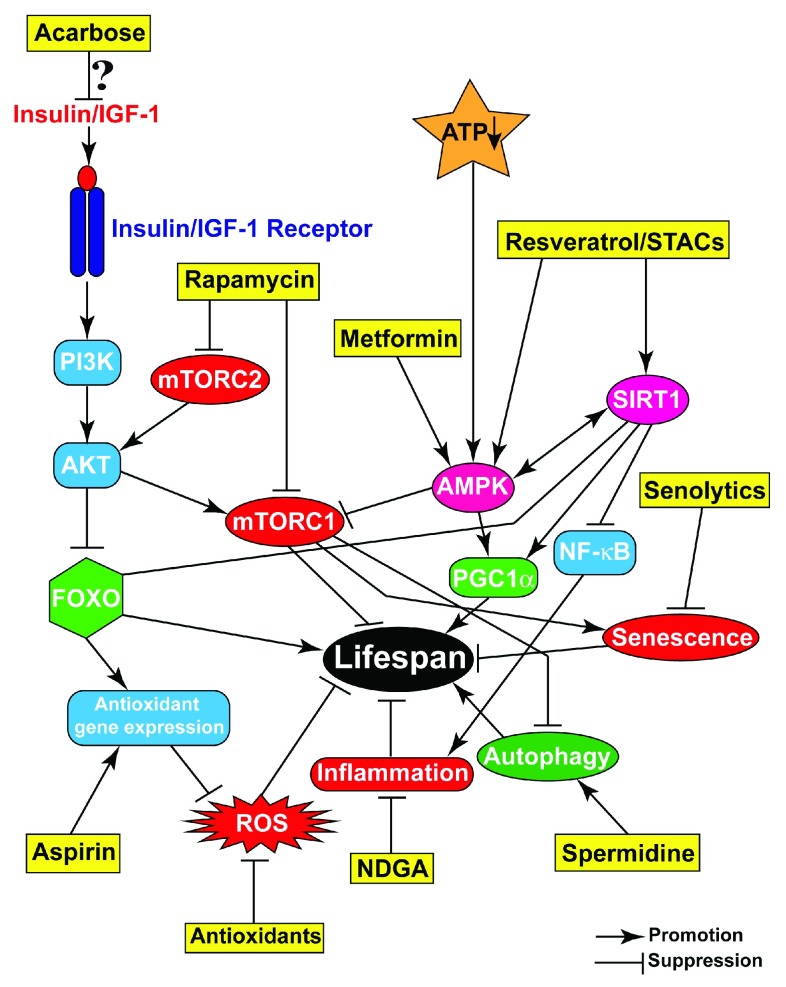
Pharmacological interventions targeting aging-related pathways and processes. Representative compounds (yellow boxes) target various processes or pathways that contribute to aging and either promote or suppress their activities/progression, resulting in improved health and enhanced lifespan.

In recent decades, numerous compounds with pro-healthspan and -longevity effects have been identified. Due to space limitations, we restrict our discussion to a few key small molecules that have shown beneficial effects, from invertebrate models to mice ( Figure 2).

Spermidine is a member of the polyamine family, involved in numerous critical cellular processes including DNA stability, transcription, translation, apoptosis, cell proliferation, and cell growth ^179^. In multiple organs, levels of polyamines have been reported to decline with age ^180, 181^. Indeed, a study by Pucciarelli *et al*. suggested that maintaining high levels of spermidine during aging might promote longevity ^182^. Administration of exogenous spermidine extended the lifespan of yeast, flies, worms, and cultured human peripheral blood mononuclear cells ^183^. Spermidine also reduces the age-related decline of locomotor performance in flies ^184^. Furthermore, it has been reported that a polyamine-rich diet reduced age-related pathology and increased lifespan in Jcl:ICR male mice ^185^. Conversely, depletion of endogenous spermidine by genetic manipulation of the polyamine pathway shortens lifespan in yeast ^183^ and mice ^186^. Spermidine supplementation reduces levels of age-related oxidative damage in mice ^183^ and also increases stress resistance in yeast ^183^ and flies ^187^. The beneficial effects of spermidine are mediated mainly via induction of autophagy ^183, 187^, allowing the regulated degradation and recycling of dysfunctional cellular components ^188^. Defective autophagy prevented the onset of spermidine supplementation-associated benefits ^183, 187^.

Aspirin, a derivative of salicylic acid, is the prototypical cyclooxygenase inhibitor and non-steroidal anti-inflammatory agent ^189^. Aspirin is a versatile drug, with antithrombotic and antioxidant properties ^190, 191^. Indeed, chronic aspirin use in humans reduces the risk of mortality from a variety of age-associated diseases, including atherosclerosis, diabetes, and a variety of cancers ^192– 196^. Aspirin use has been reported to be associated with increased survival in extreme old age in humans ^197^. In a recent study by Ayyadevara *et al.*, aspirin was shown to upregulate the expression of antioxidant genes (superoxide dismutase, catalases, and glutathione-S-transferases), resulting in attenuation of endogenous ROS levels and extension of *C. elegans* lifespan ^198^. Another study showed that aspirin treatment leads to lifespan extension in the cricket *A. domesticus* ^96^. In studies by the ITP, aspirin treatment (21 mg/kg diet) led to an increase in the mean lifespan of male mice, but there was no effect in females ^199^.

Nordihydroguaiaretic acid (NDGA), also known as masoprocol, is a naturally occurring dicatechol, with antioxidant, antiviral, antineoplastic, and anti-inflammatory activities ^200^. It has been reported to be a potent antagonist of the inflammatory cytokine TNFα. Dietary administration with NDGA delayed motor deterioration in a mouse model of amyotrophic lateral sclerosis and significantly extended lifespan ^201^. Consistently, the ITP reported that NDGA (2500 mg/kg diet) increased the lifespan of UM-HET3 male mice ^199, 202^. Lifespan extension by NDGA was not observed in female mice, even at a dose that produced blood levels equivalent to those in males ^202^. One possible explanation for this sex discrepancy could be that male controls in this study showed a somewhat short lifespan at two of the three ITP testing sites ^202^. Additional studies will be required to fully address this issue.

Acarbose is an inhibitor of α-glucosidases, intestinal enzymes that convert complex carbohydrates into simple sugars to facilitate their absorption ^203^. Acarbose treatment thus impairs carbohydrate digestion and inhibits the normal postprandial glucose rise ^203^. The ITP found that acarbose administration (1000 mg/kg diet) induced a significant increase in median and maximal lifespan in both sexes, although the impact was much more pronounced in males ^202^. Acarbose treatment increased male median lifespan by 22% (p<0.0001), but female median lifespan by only 5% (p=0.01). Similarly, maximum lifespan extension in males and females was 11% (p<0.001) and 9% (p=0.001), respectively ^202^. Acarbose-treated mice had a significant increase in levels of serum fibroblast growth factor 21 (FGF21) and also a mild reduction in IGF1 levels ^202^. FGF21 plays important roles in the regulation of glucose, lipid, and energy homeostasis ^204^. Transgenic mice with constitutive FGF21 secretion displayed an increase in both mean and maximal lifespan, probably occurring via reduced IIS ^205, 206^.

17-α-estradiol is a non-feminizing estrogen, with reduced binding affinity for estrogen receptors ^202^. It inhibits the activity of the enzyme 5α-reductase, responsible for the reduction of testosterone to the more potent androgen dihydrotestosterone ^207^, which has higher affinity for the androgen receptor than does testosterone ^208^. 17-α-estradiol has been reported to be neuroprotective against cerebral ischemia, Parkinson’s disease, and cerebrovascular disease ^209– 211^. Recently, it has been shown to diminish metabolic and inflammatory impairment in old male mice by reducing calorie intake and altering nutrient sensing and inflammatory pathways in visceral white adipose tissues, without inducing feminization ^212^. In ITP studies, administration of 17-α-estradiol (4.8 mg/kg diet) from 10 months of age increased male median lifespan by 12%, without significant effect on maximum lifespan or effects on female lifespan ^202^. Similar to NDGA, the relatively short lifespan of male controls might contribute to this apparent sex discrepancy ^202^ and further longevity studies are warranted using this drug.

β-adrenergic receptor (β-AR) antagonists bind to β-ARs (β1, 2, and 3-AR) and block the action of the endogenous catecholamines epinephrine and norepinephrine. Increased activity of β-ARs may hasten the development of age-related pathologies and increase mortality in genetically modified mice ^213– 218^. Consistently, chronic administration of β-AR agonists leads to increased mortality and morbidity ^219^. In humans, increased production of β2-AR due to specific genetic variants is associated with reduced lifespan ^220^. Conversely, dietary administration of β-AR blockers metoprolol (1.1 g/kg in the diet) and nebivolol (0.27 g/kg in the diet) increased the median lifespan of C3B6F1 male mice by 10% (p=0.016) and 6.4% (p=0.023), respectively, without affecting food intake or utilization ^221^. However, no effect was observed on maximal lifespan. Consistently, treatment with metoprolol (5 mg/mL diet) and nebivolol (100 μg/mL diet) extended the median lifespan of *Drosophila* by 23% (p≤0.0001) and 15% (p≤0.001), respectively, without impact on food intake or locomotion ^221^. Similar to β-AR blockers, an α1-AR antagonist, doxazosin mesylate, which inhibits the binding of norepinephrine to α1-AR on the membrane of vascular smooth muscle cells, extends *C. elegans* lifespan by 15% ^222^. Given that some of these agents are routinely administered clinically as antihypertensives and their safety profiles are well characterized, they may warrant further evaluation in humans specifically for their potential anti-aging effects.

Antioxidants, compounds conferring resistance to oxidative stress, have in some cases also proven successful in increasing lifespan, particularly in lower organisms. Dietary supplementation with the glutathione precursor N-acetylcysteine (NAC) increased resistance to oxidative stress, heat stress, and UV irradiation and significantly extended both the mean and the maximum lifespan of *C. elegans* ^223^ and *D. melanogaster* ^224^. Furthermore, treatment with EUK-134 and EUK-8, small molecule synthetic catalytic mimetics of superoxide dismutase (SOD) and catalase, was reported to extend *C. elegans* lifespan ^225^; however, as discussed by Gems and Doonan, other groups have not observed this effect ^226^. Treatment of a mixed group of male and female C57BL/6 mice with another SOD mimetic, carboxyfullerene (C3, at 10 mg/kg/day), reduced age-associated oxidative stress and mitochondrial superoxide production and modestly extended mean lifespan ^227^. Consistently, oral administration of carboxyfullerene (C60; 4 mg/kg/day) dissolved in olive oil to male Wistar rats leads to a 90% increase in median lifespan as compared to water-treated controls ^228^. Similarly, some other studies have shown an ability of antioxidants to extend lifespan in multiple organisms ^229, 230^.

Conversely, there are many reports that do not support the idea that dietary supplementation with antioxidants can increase the lifespan of healthy animals or humans as a general rule. Dietary supplementation with either vitamin E (α-tocopherol) or vitamin C (ascorbic acid) significantly shortened the lifespan of short-tailed field voles ^231^. Similarly, treatment of male mice with a nutraceutical mixture enriched in antioxidants was ineffective in extending lifespan ^232^. Moreover, as described in a recent review by Bjelakovic *et al.*, systematic review and meta-analyses of a large number of randomized clinical trials evaluating the effects of dietary supplementation with various anti-oxidants (β-carotene, vitamin A, vitamin C, vitamin E, and selenium) in humans did not reveal any overall benefit; indeed, in some cases, there was evidence for increased mortality occurring in response to these agents ^233^. Deleterious effects of antioxidant supplementation may result from inappropriate suppression of the normal signaling functions ROS play in cells, including in crucial cell populations such as stem cells ^234^.

### Selective deletion of senescent cells by senolytic drugs

Cellular senescence refers to permanent cellular growth arrest, which can be induced by multiple stressors, including serial passage, telomere attrition, inappropriate mitotic stimuli, and genotoxic insult ^235^. Senescence is thought to play an important role in tumor suppression in mammals ^236, 237^. However, senescent cells develop an altered secretory phenotype (termed the SASP) characterized by the release of factors such as proteases, growth factors, interleukins, chemokines, and extracellular remodeling proteins ^238^. With advancing age, senescent cells accumulate in various tissues ^239– 241^ and potentially contribute to pathological states, as factors they secrete induce chronic inflammation, loss of function in progenitor cells, and extracellular matrix dysfunction ^236, 242^. The functional impact of senescent cells *in vivo* has been a hotly debated topic in aging biology for many years. Recently, genetic approaches to delete senescent cells in mice have been described, via activation of a drug-inducible “suicide gene” ^243^. Depleting senescent cells in a progeroid mouse model substantially delayed the onset of multiple age-related phenotypes, including lordokyphosis (a measure of sarcopenia in this model), cataract, loss of adipose tissue, and impaired muscle function ^243^. However, the overall survival of these mice was not extended substantially by deletion of senescent cells, perhaps because the suicide gene was not expressed in the heart or aorta; cardiac failure is thought to represent a major cause of mortality in this strain ^243^. A recent landmark study by Baker *et al.* showed that clearance of naturally occurring senescence cells in non-progeroid mice maintained the functionality of several organs with age, delayed lethal tumorigenesis, and extended median lifespan in mixed and pure C57BL/6 genetic backgrounds by 27% (p<0.001) and 24% (p<0.001), respectively ^244^. This study provides very strong evidence that age-associated accumulation of senescent cells contributes to age-associated pathologies and shortens lifespan in WT animals.

Pharmacologic, as opposed to genetic, approaches to deplete senescent cells have posed a major technical and conceptual challenge. A recent study showed that senescent cells display increased expression of pro-survival factors, responsible for their well-known resistance to apoptosis ^245^. Interestingly, small interfering RNA (siRNA)-mediated silencing of many of these factors (ephrins, PI3Kδ, p21, BCL-xL, and others) selectively killed senescent cells but left dividing and quiescent cells unaffected. These siRNAs were termed “senolytic” siRNAs ^245^. Small molecules (senolytic drugs) targeting the same factors also selectively killed senescent cells. Out of 46 agents tested, dasatinib and quercetin were particularly effective in eliminating senescent cells. Dasatinib, used in cancer treatment, is an inhibitor of multiple tyrosine kinases ^246^. Quercetin is a natural flavonol that inhibits PI3K, other kinases, and serpins ^247, 248^. Dasatinib preferentially eliminated senescent human preadipocytes, while quercetin was more effective against senescent human endothelial cells and senescent bone marrow-derived murine mesenchymal stem cells (BM-MSCs). The combination of dasatinib and quercetin was effective in selective killing of senescent BM-MSCs, human preadipocytes, and endothelial cells ^245^. The combination was more effective in killing senescent mouse embryonic fibroblasts compared to either drug alone. Treatment of chronologically aged WT mice, radiation-exposed WT mice, and progeroid *Ercc1* hypomorphic mice with the combination of dasatinib and quercetin reduced the burden of senescent cells. Following drug treatment, old WT mice showed improved cardiac function and carotid vascular reactivity, irradiated mice displayed improved exercise capacity, and progeroid *Ercc1 ^-/Δ^* mutants demonstrated delay of age-related symptoms and pathologies ^245^. Similarly, a recent study by Chang *et al.* identified ABT263 (Navitoclax, a specific inhibitor of the anti-apoptotic proteins BCL-2 and BCL-xL) as another potent senolytic agent ^249^. ABT263, which is used for the treatment of multiple cancers ^250– 252^, induced apoptosis and selectively killed senescent cells in a manner independent of cell type or species ^249^. In culture, senescent human lung fibroblasts (IMR90), human renal epithelial cells, and mouse embryo fibroblasts (MEFs) were more sensitive to ABT263 treatment than their non-senescent counterparts ^249^. In contrast, another study found that ABT263 is not a broad-spectrum senolytic; instead it acts in a cell type-specific manner ^253^. In this study, ABT263 was found to be senolytic in human umbilical vein cells (HUVECs), IMR90 cells, and MEFs, but not in human primary preadipocytes ^253^.

Treatment of either irradiated or naturally aged mice with ABT263 not only reduced the burden of senescent cells, including those among bone marrow hematopoietic stem cell (HSC) and muscle stem cell (MuSC) populations, but also suppressed the expression of several SASP factors and rejuvenated the function of aged HSCs and MuSCs ^249^. These results, together with the impressive results obtained in genetic models described previously, indicate that senolytic drugs may have a role in improving tissue function during aging. However, some senolytic drugs are associated with toxic side effects, like thrombocytopenia and neutropenia in the case of ABT263, which are major potential hurdles in their use as anti-aging therapies. These toxicities may be mitigated somewhat if these drugs can be administered intermittently, rather than chronically, to achieve their senolytic effects.

Major results concerning the small molecules discussed in this review are summarized in Figure 2.

## From model organisms to humans: the challenges of screening for anti-aging drugs

Several drugs have demonstrated great promise in the laboratory setting in enhancing the healthspan and lifespan of multiple species, including mice, raising the possibility that efficacious pharmacologic anti-aging therapy in people may be possible. However, screening for novel small molecules with anti-aging effects in mammals in an unbiased fashion represents an enormous, potentially insurmountable challenge. Alternatively, since it is clear that several cellular pathways affect longevity in an evolutionarily conserved manner, invertebrate models may be quite useful for such screening endeavors. However, some known molecular factors with major effects on mammalian lifespan ( *e.g.* GH) are not well conserved between invertebrates and mammals. Consequently, small molecule screening efforts relying exclusively on the use of invertebrates will likely miss drugs with potent effects on mammalian aging. Moreover, many of the key physiologic features of humans and other mammals are not well modeled in invertebrates, as the latter lack specific tissues like heart and kidney and complex endocrine, nervous, and circulatory systems that are crucial targets of mammalian aging and age-related pathologies. Most invertebrate aging models possess limited regenerative capabilities and incompletely recapitulate processes such as stem cell renewal, which are required for tissue repair mechanisms that maintain tissue homeostasis in mammals, in order to sustain organ function over years and decades.

The development of new, shorter-lived vertebrate aging systems could be tremendously beneficial in screening for drugs with anti-aging activities. In this context, several features of the naturally short-lived vertebrate African turquoise killfish ( *N. furzeri*) make this organism an attractive model system to study various aspects of vertebrate aging and potentially as a drug-screening system ^254– 258^. Recently, using a *de novo*-assembled genome and CRISPR/Cas9 technology, Harel *et al.* described a genotype-to-phenotype platform in *N. furzeri*, opening up the possibility of screening for gene mutations and drugs that increase lifespan in this organism in an integrative fashion ^259^. One current major limitation of *N. furzeri* is the need for individual housing in aging studies, greatly increasing husbandry costs. Moreover, it is possible that some of the factors modulating aging in fish and other cold-blooded vertebrates may be dissimilar to those in mammals.

Although mice faithfully recapitulate many aspects of human aging and age-associated diseases, their use in primary screening/testing of a large number of potential anti-aging compounds is not feasible because of the high associated costs. The use of progeroid models, such as *Ercc1* hypomorphs or *Lmna* mutants, with accelerated pathology and short lifespan, might allow the evaluation of many more compounds than could be reasonably tested in WT mice ^260, 261^; however, whether or not such animals suffer from aging *per se* is a hotly debated topic ^262, 263^. Likewise, it is possible that rigorous delineation of appropriate surrogate markers of aging – *e.g.* increased p16 expression ^264^ or altered DNA methylation (DNAm) ^265^ – may allow initial evaluation of a large number of compounds in mice for potential anti-aging effects, without the need to perform costly and lengthy lifespan studies on many different cohorts, each treated with different candidate anti-aging compounds. In this regard, the Horvath group has developed an approach that allows estimation of the age of most tissues and cell types based on age-associated alterations in DNAm levels at 353 CpG sites ^266^. To the author’s knowledge, longevity screens using surrogate markers such as DNAm have not been attempted in mice.

To date, the discovery of anti-aging compounds has so far been carried out via two basic approaches. One of these is phenotypic, defined as the screening of compounds in cellular or animal models to identify drugs conferring desired biological effects, *i.e.* lifespan extension ^267, 268^. Although this approach has proven enormously valuable in many areas of biochemical research, identifying drugs that can modulate lifespan is more time consuming, complex, and expensive than for many other phenotypes ^267, 268^. Moreover, elucidating the mechanism of action of agents identified in such phenotypic, “black box” screens represents a formidable challenge, though the powerful genetic tools available in invertebrate models can facilitate such efforts. One currently underutilized system with respect to small molecule-based longevity screens is the budding yeast, *S. cerevisiae.* Two distinct forms of aging have been characterized in this organism, replicative and chronological (population based) ^269^. In principle, either might serve as the basis for screens for anti-aging compounds, though chronological aging is far more amenable to high-throughput analysis. A complementary approach involves target-based screening for modulators of pathways known or strongly suspected to modulate the aging rate ^267^. However, by definition, such efforts are unlikely to identify novel cellular factors and pathways involved in longevity.

**Figure 3.**
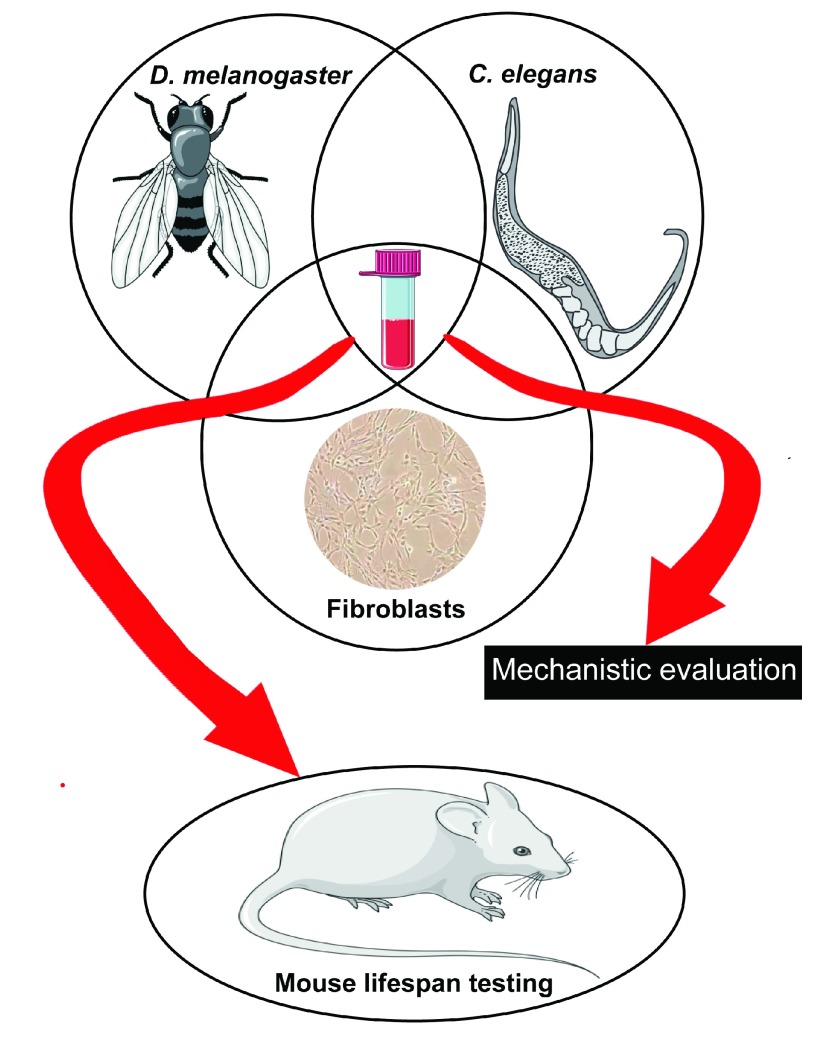
Approach being followed at University of Michigan for identification of compounds with potential anti-aging effects. Drugs identified for their ability to increase healthspan and lifespan in *Drosophila* and *Caenorhabditis elegans* and to enhance stress resistance in mammalian fibroblasts are potential candidates for further in-depth mechanistic evaluation and testing in mice.

To address these complications, a holistic approach, involving complementary efforts in invertebrates, mammalian cells, and mice, might represent a powerful combination in the quest for anti-aging compounds. With the important caveats noted above, invertebrates can be efficiently used for primary screening of thousands of compounds to identify a few selected candidates with potential anti-aging effects for further testing in mice. In this context, in our Center ( http://www.med.umich.edu/geriatrics/research/glenn/), supported by the Glenn Foundation for Medical Research, compounds are screened for their ability to increase healthspan and lifespan in *Drosophila* and *C. elegans* and for enhancement of stress resistance in mammalian fibroblasts, a correlate of longevity in mammals ^270^. Compounds that are efficacious in all of these assays are candidates for more in-depth mechanistic evaluation and for further testing in mice ( Figure 3).

A related challenge in aging research at present is the lack of primate model systems with reasonably short lifespan for preclinical testing of candidate anti-aging drugs. The most commonly used model, the rhesus monkey, lives for three to four decades ^20^. Another primate, the common marmoset, has several advantages over rhesus monkeys in terms of size, availability, and other biological characteristics ^271^. Because of their small size, marmosets generally cost less to feed and house in comparison with the rhesus monkey. Furthermore, the marmoset has a gestation period of ~147 days and usually gives birth to 2–3 offspring per delivery. Some marmoset traits more closely resemble those of humans than do those of rhesus, including their disease susceptibility profile. In Europe, the marmoset is used as a non-rodent species for drug safety assessment and toxicology ^271^. In this regard, in a recent report, Tardif *et al.* described the dosing procedure, pharmacokinetics, and downstream signaling changes for rapamycin administration to marmosets ^272^. However, their maximal lifespan is ~17 years – shorter than the rhesus monkey, but still highly impractical for testing pharmacological interventions aimed at extending longevity. The development of new mammalian aging models besides the mouse would be extremely helpful to better elucidate the biological processes underlying mammalian aging and to expedite the translation of pharmacological interventions from the laboratory to actual clinical use in humans.

One model to consider in this regard is dogs, which share their social environment with humans ^273^. Furthermore, dogs are relatively well understood with regard to aging and disease, exhibit great heterogeneity in body size and lifespan, and provide a large pool of genetic diversity. Dogs might represent a relatively inexpensive model system, particularly if some dog owners were willing to test candidate lifespan-extending drugs that had previously been validated in invertebrate and rodent models. Indeed, identifying interventions that can promote healthspan and lifespan in dogs may represent an excellent entrée to achieving the same goals in humans. In this context, Matthew Kaeberlein and Daniel Promislow at the University of Washington in Seattle have launched a pilot trial involving 30 dogs aimed at testing the efficacy of rapamycin in improving overall health and extending lifespan in large dogs that usually survive for 8 to 10 years ^274^.

Testing candidate anti-aging compounds in humans represents an enormous challenge ^112^. It is highly unlikely that pharmaceutical companies can be persuaded to engage in decades-long clinical trials of candidate anti-aging medicines with lifespan as an endpoint. The evaluation of shorter-term surrogate phenotypes, such as molecular markers or age-associated defects such as impaired responses to vaccination ^75^, may permit initial clinical evaluation of candidate anti-aging compounds in a more reasonable timeframe.

## Conclusion

Since ancient times, humanity has dreamed of interventions to slow the aging process and prolong lifespan. However, only in the modern era has biological aging research progressed to the point where interventions that delay human aging may eventually represent a real possibility. Accumulating work in invertebrate models and rodents has identified an ever-growing list of molecules with the ability to extend lifespan and promote late-life health in mammals. Given the intimate link between aging and disease, such drugs may dramatically improve human health if the major challenges in their testing and deployment can be overcome.
